# Chemically Modified Biomimetic Carbon-Coated Iron Nanoparticles for Stent Coatings: In Vitro Cytocompatibility and In Vivo Structural Changes in Human Atherosclerotic Plaques

**DOI:** 10.3390/biomedicines9070802

**Published:** 2021-07-12

**Authors:** Shamil Akhmedov, Sergey Afanasyev, Marina Trusova, Pavel Postnikov, Yulia Rogovskaya, Elena Grakova, Kristina Kopeva, Rosa Karen Carreon Paz, Sascha Balakin, Hans-Peter Wiesmann, Joerg Opitz, Benjamin Kruppke, Natalia Beshchasna, Sergey Popov

**Affiliations:** 1 Tomsk National Research Medical Center of the Russian Academy of Sciences, Cardiology Research Institute, 634012 Tomsk, Russia; Tursky@cardio-tomsk.ru (S.A.); pathan@cardio-tomsk.ru (Y.R.); gev@cardio-tomsk.ru (E.G.); kristin-kop@inbox.ru (K.K.); psv@cardio-tomsk.ru (S.P.); 2 Research School of Chemistry and Applied Biomedical Sciences, Tomsk Polytechnic University, 634050 Tomsk, Russia; postnikov@tpu.ru; 3 Fraunhofer Institute for Ceramic Technologies and Systems IKTS, 01109 Dresden, Germany; rosa_karen.carreon_paz@tu-dresden.de (R.K.C.P.); sascha.balakin@ikts.fraunhofer.de (S.B.); joerg.opitz@ikts.fraunhofer.de (J.O.); 4 Max Bergmann Center of Biomaterials, Institute of Materials Science, Technische Universität Dresden, 01069 Dresden, Germany; wiesmann@msx.tu-dresden.de (H.-P.W.); Benjamin.kruppke@tu-dresden.de (B.K.)

**Keywords:** atherosclerosis, high density lipoprotein, chemically modified carbon-coated iron nanoparticle, reverse cholesterol transport, macrophage, coronary stent

## Abstract

Atherosclerosis, a systematic degenerative disease related to the buildup of plaques in human vessels, remains the major cause of morbidity in the field of cardiovascular health problems, which are the number one cause of death globally. Novel atheroprotective HDL-mimicking chemically modified carbon-coated iron nanoparticles (Fe@C NPs) were produced by gas-phase synthesis and modified with organic functional groups of a lipophilic nature. Modified and non-modified Fe@C NPs, immobilized with polycaprolactone on stainless steel, showed high cytocompatibility in human endothelial cell culture. Furthermore, after ex vivo treatment of native atherosclerotic plaques obtained during open carotid endarterectomy surgery, Fe@C NPs penetrated the inner structures and caused structural changes of atherosclerotic plaques, depending on the period of implantation in Wistar rats, serving as a natural bioreactor. The high biocompatibility of the Fe@C NPs shows great potential in the treatment of atherosclerosis disease as an active substance of stent coatings to prevent restenosis and the formation of atherosclerotic plaques.

## 1. Introduction

Inventions in nanotechnology widen the scope of the approaches for the development of new medical technologies, including those for atherosclerosis treatment. The process of atherosclerotic plaque formation and the accompanying molecular and cellular events create numerous opportunities for affecting them using nanoparticles (NPs) [1]. By attaching antibodies, proteins, peptides, or other ligands to their surfaces, NPs can be targeted to single or multiple receptors that are expressed on the surface or inside of an atherosclerotic plaque [1,2,3,4]. Different processes, markers, and cells have been manipulated using NPs to achieve desired therapeutic effects [5].

Metallic NPs, such as iron oxide NPs with a size of 15–60 nm, usually comprise a category of paramagnetic agents that can be coated with different compounds to inhibit their aggregation and enhance their stability in order to be used as a passive or active targeting agent for recognition and localization of disrupted plaques [6]. The research group around R. Weissleder developed MRI agents to image plaque components called “magnetic switches”, made from iron oxide particles that contain many copies of high-affinity ligands used to detect the activity of myeloperoxidase in plaques, which is an enzyme that has been suspected of being a product of inflammatory cells that incite plaque instability and that may lead to myocardial infarction [7]. The group of Frias et al. reported the development of recombinant paramagnetic HDL-like particles that enhance atherosclerotic regions in apolipoprotein E (ApoE)-deficient mice. They were composed of normal isolated human HDL-particles and reconstituted phospholipids with a phospholipid-base of conjugated Gd-pentetic acid (DTPA) for signal improvement, in their study nonselective accumulation of the particles in atherosclerosis was demonstrated [8].

Utilizing the natural ability of HDL molecules to promote macrophage cholesterol efflux and reverse cholesterol transport belonging to the most important physiological mechanisms of protection against atherosclerosis, several therapeutic strategies have been proposed [9,10]. In particular, HDL mimicking NPs have attracted special attention as a promising solution for atherosclerosis treatment and imaging. In comparison to other types of NPs used in combination with anti-atherosclerotic drugs or targeting ligands, drug-free HDL mimicking NPs demonstrate intrinsic anti-atherosclerotic activity and targeting ability for atherosclerotic plaques [10].

The key issue in the application of nanoparticles in the field of medicine is their biological inertness and the possibility of surface functionalization to create necessary properties. Fe@C NPs satisfy these demands [11] and can be easily modified through the use of arendiazonium salts [12,13]. The present study aims to mimic the natural structure of lipoproteins [14] by using chemically modified Fe@C NPs and investigate their interactions with human atherosclerotic plaques under in vivo and in vitro conditions. The results of morphometric and histological analysis, and electron microscopy images obtained through investigation of human atherosclerotic plaques treated by Fe@C NPs after 21 days of implantation in Wistar rats, are discussed; the Fe@C NPs demonstrated an ability to penetrate into atherosclerotic plaque structures and cause structural changes. To confirm this effect, further studies, e.g., in vivo experiments under flowing blood flow and including histological and immunohistochemical analyses, as well as experiments with macrophages in culture should be carried out as an extension of this study, and are proposed as a basis for further research.

## 2. Materials and Methods

### 2.1. Materials Preparation of Chemically Modified Carbon-Coated Iron Nanoparticles

**Figure 1:**
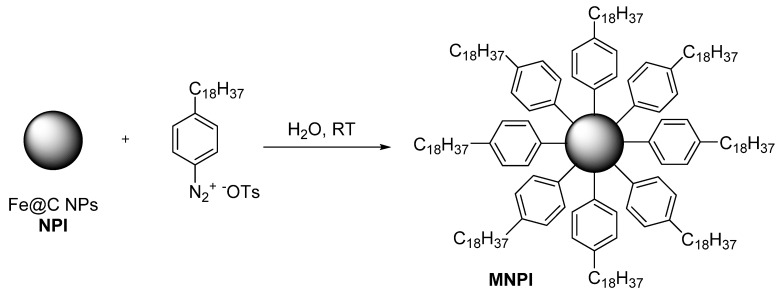
The surface modification of Fe@C NPs by lipophilic organic groups mimicking atheroprotective biomimetic HDL.

The carbon-coated iron nanoparticle Fe@C with a size of ~10 nm were obtained by gas-phase synthesis, as described earlier [13]. Fe@C NPs were modified with 4 octadecyl benzene diazonium tosylate, prepared according to the published procedure (Figure 1) [15]. Briefly, a solution of 4-octadecyl benzene diazonium tosylate (0.01 mol) in 5 mL of H_2_O/MeOH mixture (1:1 *v*/*v*) was added to 0.03 g of Fe@C that had been preliminarily dispersed in 5 mL of distilled water (ultrasonic radiation at 22.2 kHz in 2 s). The reaction mass was held for 10 min with periodic agitation. The modified nanoparticles were separated using a magnet and washed twice with water, ethyl alcohol, and acetone. Acetic acid (reagent grade, ≥99%), diethyl ether, deionized water, 4-octadecylaniline (97%), p Toluenesulfonic acid monohydrate (ACS reagent, ≥98.5%), and high-purity water (EMD Millipore) were purchased from Sigma-Aldrich and used without further purification.

### 2.2. Fe@C NP Immobilization

For cell culture experiments, the Fe@C NPs were immobilized on stainless steel discs using polycaprolactone (PLA) and a spin coating technique. Before spin coating, all stainless-steel discs (medical grade 316L), 10 mm in diameter and 2.5 mm in thickness, were ground with SiC paper (400, 600, and 1000 grit), ultrasonically cleaned in acetone, ethanol, and ddH_2_O for 5 min, and dried with N_2_. The solutions and the coating procedures are based on the literature; PLA powder was weighed and dissolved in dichloromethane (DCM) to obtain 10% (*w*/*v*) solutions, stirred at 400 rpm, and heated at 60 °C for 30 min [16,17,18]. Following the formation of a jelly-like material, nanoparticles were gradually added. Finally, the mixture was magnetically stirred continuously for 10 min, followed by ultrasonic dispersion for 5 min. Six solutions were prepared using non-modified and modified Fe@C NPs at concentrations of 3, 10, and 20 wt.% each. For spin coating, 500 µL of the PLA-nanoparticle suspension was applied to the stainless-steel discs using a micropipette and spin-coated for 30 s at a rotating speed of 2000 rpm. Afterward, the sample was immediately dried in air at room temperature leading to a one-layer coating.

### 2.3. In Vitro Cell Culture of Human Umbilical Vein Endothelial Cells

Cytocompatibility of the coatings was evaluated using cell culture experiments with human umbilical vein endothelial cells (HUVEC). Three samples were used for each cell culture with stainless steel discs and pure PLA coatings on steel discs serving as references. All samples were sterilized by autoclaving at 121 °C for 30 min prior to use for cell cultures. The samples were placed in 24-well plates and seeded with 50 µL HUVEC cells at a density of about 20,000 cells in 500 µL endothelial cell basal medium (containing endothelial cell supplement pack and 1% penicillin–streptomycin). Furthermore, cells were seeded directly on polystyrene well plates at the same density as the reference and a calibration curve for HUVEC cells was obtained by seeding 200,000, 100,000, 50,000, 25,000, 12,500, 5000, 2500, and 0 cells. Samples were incubated for one, three, and seven days at 37 °C with 5% CO_2_. The medium was changed on days one and four. After each incubation period, the medium was removed and stored in Eppendorf tubes for further analyses. The samples were washed with phosphate-buffered saline (PBS) to remove non-adherent cells and placed in new well plates. The empty well plates and the well plates containing the samples were frozen for further analyses. All tests were performed in triplicates.

### 2.4. Analysis of HUVECs after Culture on Coatings of Fe@C in PLA

The number of cells was measured by DNA quantification with Pico-Green (Quant-iT^TM^ PicoGreen^®^dsDNA Reagent, Sigma-Aldrich, Darmstadt, Germany). The samples were lysed with 550 µL 1% TritonX-100 (Sigma-Aldrich) in PBS and left on ice for 1 h with continuous agitation. A total of 10 µL of the cell lysates, as well as three blanks of 10 µL of 1% Triton X-100, were pipetted into black 96-well plates. A solution consisting of 1× TE buffer and 1:800 Pico-Green was prepared and 190 µL of this solution was added to each well. Each well’s fluorescence was measured on an Infinite 200 Pro TECAN plate reader (Tecan, Männedorf, Switzerland) with standard fluorescence filter at a 485-nm excitation and 535-nm emission. The final cell number of each sample was calculated using a HUVEC standard curve.

Furthermore, to detect cell adherence to the samples’ surfaces and to judge their morphology, immunofluorescence staining of HUVECs (after seven days of culture) was performed. Cells were fixed using 3.7% formaldehyde in PBS for 30 min at 4 °C, washed with cold PBS, and then 0.37% formaldehyde was added and left overnight at 4 °C. Before staining, the fixed cells were permeabilized with 0.2% Triton X-100 in PBS for 5 min and washed three times with PBS for 2 min to remove any excess. The staining solution was prepared in bovine serum albumin (BSA) containing DAPI (4′,6-diamidino-2-phenylindole) at 1:500 and Alexa Fluor™ 488 Phalloidin (life technologies, Thermo Fisher Scientific, Waltham, MI, USA) at 1:50. Samples were covered with the staining solution for 1 h and protected from light. Subsequently, they were washed four times with PBS for 2 min, stored in PBS at 4 °C, and protected from light. Confocal microscopy of the stained HUVECs was performed using a LSM 510 META by Carl Zeiss (Jena, Germany) with a 20× objective.

### 2.5. Preparation of Human Atherosclerotic Plaques and In Vivo Experiment

The Fe@C NPs were used in their initial powdery state. Samples of atherosclerotic plaques were obtained during routine open carotid endarterectomy surgery.

Carotid endarterectomy operations were performed in 20 male patients aged 62 ± 1.2 years, in whom an extended atherosclerotic plaque, according to ultrasound examination, narrowed the lumen of the internal carotid artery by 75% None of them took cholesterol-lowering drugs on an ongoing basis.

Forty square samples (1 cm × 1 cm) containing atherosclerotic plaques were prepared. The samples were dampened in physiological solution, half of them (*n* = 20) were uniformly dusted with sterile powder of chemically modified Fe@C NPs, while for the second half of the samples, non-modified Fe@C NPs were used. After the treatment with Fe@C NPs, atherosclerotic plaques were formed into a round shape for compact subcutaneous implantation into animals.

The animal experiments were performed in accordance with the ethical standards laid down in the WMA Statement on Animal Use in Biomedical Research (2006) and the Guide for the Care and Use of Laboratory Animals of the Research Institute of Cardiology, Tomsk National Research Medical Center, the Russian Academy of Science.

Fifty laboratory animals were used for the experiments. For each checkpoint, there were 5 rats, which included the control group, as well as plaque-treated with modified and unmodified nanomaterials. Studies of the implanted plaques were performed after 7, 14, and 21 days, respectively.

The design and current research protocol were approved by the ethics committee of the Research Institute of Cardiology, Tomsk National Research Medical Center, the Russian Academy of Science (Corresponding ethical approval protocol no. 112 from 23 October 2013). In vivo tests were carried out on laboratory Wistar rats (male) with a weight range of 200 g to 250 g. The prepared atherosclerotic plaques, with and without Fe@C treatment, were implanted under the skin of animals under sterile conditions and anesthesia using a routine method with narcotized ketamine (50 mg/kg intraperitoneally) [19]. Subcutaneous implantation was performed by making a cut on the animals’ backs. The implantation of the treated plaques into rats played a role of a bioreactor, providing in vivo conditions that were maximally close to a biological environment. After implantation, the cut was sewn up and, after waking up, the animals were treated routinely in their home cages in the vivarium. After 7, 14, and 21 days of implantation, the samples were explanted, deployed (from the cylindrical form) or cross-sectioned, subjected to morphometric analyses, and fixed for histological and electron microscopy (EM) studies. In this way, it was possible to inspect outer and inner plaque structures and evaluate the penetration of the nanomaterial into the inner plaque volumes. The maximal implantation time of 21 days was selected experimentally as the longest time during which the plaque structure could be kept live in a laboratory animal. As a basis to define the maximal implantation time, we experimentally discovered that after longer than one month of implantation, the plaques in the experimental animals lysed completely without any possibility of identify them. According to our knowledge and taking into consideration the size of the studied implanted plaques, the organism of the Wistar rat can serve as a bioreactor.

### 2.6. Analysis of Human Atherosclerotic Plaques after In Vivo Experiment

After explantation of the atherosclerotic plaques, their histological structures were studied in semi-fine sections, dyed with 1% solution of methylene blue at an optical magnification of 1000×. The areas of metachromatic staining in the matrix of atherosclerotic plaques around the chemically modified nanoparticles were measured. In this case, the total area of the nanoparticles themselves was subtracted from the total estimated area, and thus the true area of metachromatic staining was calculated (µm^2^).

Quantitative measurements of the area of metachromatic staining of the matrix of the atherosclerotic plaques around the chemically modified nanoparticles were carried out using ZEN 2 software (blue edition) using Carl Zeiss Microscopy GmbH.

The microstructure was analyzed using transmission electron microscopy (TEM) [10]. Ultrathin sections were prepared using Wikly’s method [20]; sections 60–100-nm thick were prepared using an Ultrotome III (LKB, Stockholm, Sweden), placed on an underlayer net with a formvar film, contrasted in a 2% uranyl acetate solution of 50% ethanol (for 10–20 min at 37 °C) and lead citrate (for 3–10 min at room temperature) using the Reynolds method [21]. The obtained samples were analyzed using an electronic microscope (JEM-100 CXII; JEOL, Tokyo, Japan) with an aperture diaphragm of 25–30 µm and an accelerating voltage of 80 keV.

### 2.7. Statistics

Measurements were performed in triplicate (at least) and the results are expressed as means ± standard deviation. Two-way analysis of variance (ANOVA) was performed for statistical analysis of cell count measurements with time and materials as the two parameters, and using Bonferroni post hoc test. *p* values < 0.05 were considered significant and are indicated by an asterisk.

## 3. Results

### 3.1. Cytocompatibility—HUVECs on Non-Modified and Modified Fe@C in PLA

**Figure 2:**
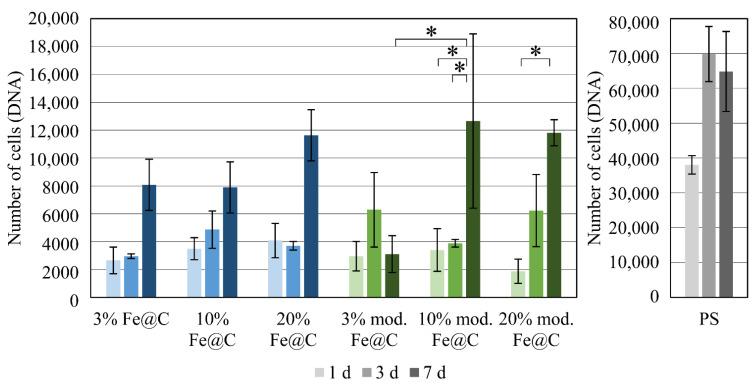
Cell counts as means ± standard deviation calculated from DNA measurements after HUVEC culture over one, three, and seven days on stainless steel coated with PLA containing 3%, 10%, and 20% non-modified Fe@C NPs (blue) and chemically modified Fe@C NPs (green). HUVECs cultured on well plate polystyrene (grey) is used as positive reference. Asterisks (*) indicate statistically significant differences (*p* < 0.05).

The Fe@C NPs were immobilized on stainless-steel samples using PLA and a spin coating technique to evaluate their cytocompatibility. HUVECs were cultured on such substrates with a count of 2 × 10^4^ cells per well. The number of cells was determined by DNA quantification with Pico-Green after one, three, and seven days of culture in combination with a standard grow curve (Figure 2). Polystyrene (PS) was used as the reference material. There was a significant difference in the time of cultivation between one day and three days, as well as one day and seven days. In general, approximately 10% of the HUVECs adhered to the Fe@C NP-modified PLA coatings compared to PS. The samples possessing 3% modified Fe@C NPs were shown to cause almost no cell proliferation. Starting from equivalent cell adherence of all materials, the lowest number of cells after seven days of approximately 3000 cells were present on 3% modified Fe@C NPs. For all the other samples, an increase in the cell count during cultivation was measured. The equal cell count levels at seven days, which were only significantly different in the case of 3% vs. 10% modified Fe@C NPs, revealed that chemical modification does not change the cytocompatibility of Fe@C NPs in PLA with respect to HUVECs.

**Figure 3:**
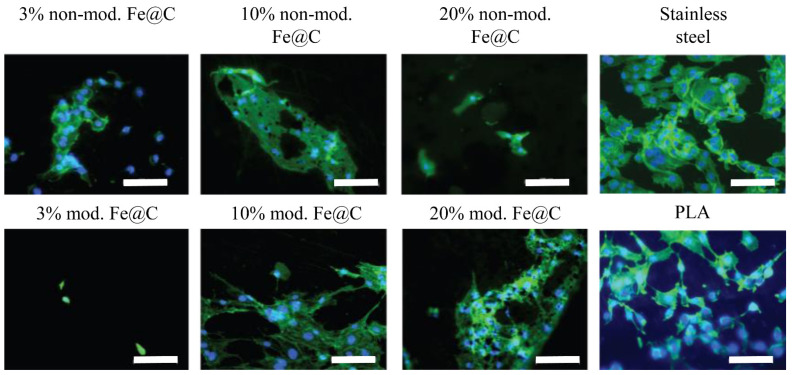
Fluorescent microscopy images of HUVECs after seven days in culture on the surface of PLA coated stainless steel samples with 3%, 10%, and 20% of non-modified and modified Fe@C, as well as directly on stainless steel and plain PLA as references. Scale bar equals 100 µm. Nuclei are stained with DAPI and depicted in blue. Cellular actin skeleton is stained with phalloidin and is visible in green.

In addition to quantitative cytocompatibility evaluation, morphological investigations of the development of HUVECs were also performed using fluorescence imaging and DNA staining to indicate the nuclei, and actin to visualize the cytoskeleton. Cells showed a high degree of aggregation, which can be seen from the wide elongated actin skeleton areas in the cases of 10% non-modified Fe@C, 10% modified Fe@C, 20% Fe@C, and the references for morphological investigation using stainless steel and PLA, respectively (Figure 3). It should be emphasized that the cells on PLA without NPs had a round morphology, while on the samples with 10% and 20% modified Fe@C NPs had a characteristically more phenotypically elongated morphology, forming a nearly porous cell network.

### 3.2. Morphometric Analysis of Plaques after Subcutaneous Implantation

Square-shaped human atherosclerotic plaque samples were treated with non-modified Fe@C NPs and chemically modified Fe@C NPs, rolled into a cylindrical shape, and implanted into Wistar rats. Morphometric analysis of the explanted plaque samples demonstrated penetration of the chemically modified Fe@C NPs into the inner structure of the plaque tissue, as well as changes to its consistency, as a result of 21 days of in vivo experimentation. The non-modified Fe@C NPs were accumulated on the plaque surface, as they were prepared, and did not cause any structural alterations to the inner part of the plaque samples.

**Figure 4:**
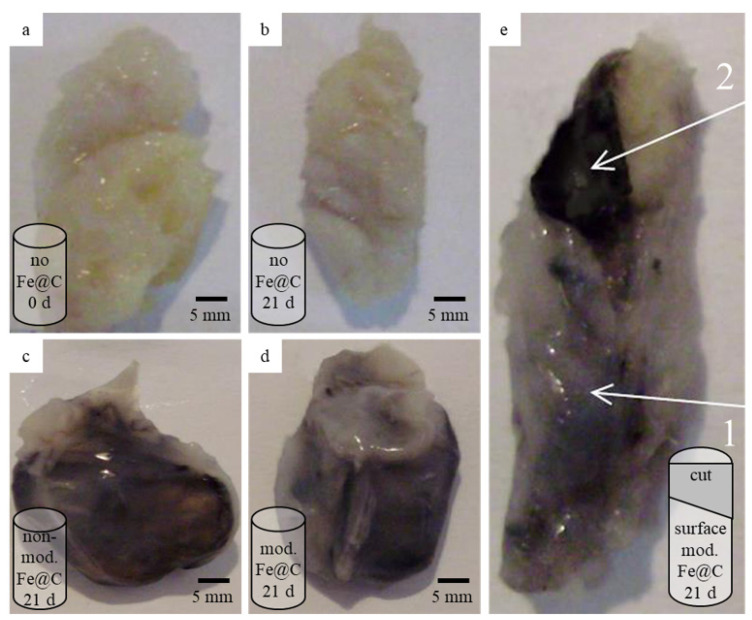
Rolled-up samples of atherosclerotic plaques used for subcutaneous implantation in rat in vivo experiments: (**a**) atherosclerotic plaque in the initial state (before implantation in rats, not treated with NPs); (**b**) atherosclerotic plaque not treated with NPs explanted after 21 days implantation in rat; (**c**) atherosclerotic plaque treated with non-modified Fe@C NPs explanted after 21 days implantation in rat; (**d**,**e**) atherosclerotic plaque treated with chemically modified Fe@C NPs explanted after 21 days implantation in rat. Schematic representation of plaques samples with depiction of cut sample in (**e**) 1—outer surface of the explanted fragment of the atherosclerotic plaque; 2—area of the cross-section, demonstrating the inner structure of the atherosclerotic plaque.

The control group of plaque samples, which were not treated with NPs, after 21 days of subcutaneous implantation into rats showed no significant morphometric changes of the atherosclerotic plaques and they retained their yellowish-white color in comparison to the initial state (Figure 4a,b). Comparing to the non-treated plaques, the plaques treated with non-modified Fe@C NPs (Figure 4c) and chemically modified Fe@C NPs (Figure 4d,e) explanted after 21 days of implantation demonstrated an atypical glossy dark color.

After surgical explanation of plaques after 7, 14, and 21 days, we did not visually observe fibrous capsules around them. We explain this by the fact that the follow-up period was relatively short and therefore fibrous encapsulations could not be formed.

The general appearance of the explanted atherosclerotic plaques, surface-treated with modified Fe@C NPs and non-modified Fe@C NPs, did not differ much visually. However, macroscopic analyses of the cross-sections of the atherosclerotic plaques confirmed the penetration of modified Fe@C NPs into the inner plaque structure, staining its fragments in grey and black tones. A buildup of black agglomerates caused by modified Fe@C NPs was observed, not only on the plaque surface, but also in the inner structure as well (as shown in Figure 4e). The penetration depth of the modified Fe@C NPs correlated with the duration of implantation. The longer the implantation duration, the deeper the penetration (pictures not shown). In the case of non-modified Fe@C NPs, no penetration into the inner structure of the plaque tissue was observed. The non-modified Fe@C NPs were accumulated on the plaque surface. The consistency of the plaque tissues treated with modified Fe@C NPs and non-modified Fe@C NPs differed from each other. The plaque tissue treated with non-modified Fe@C NPs was more consistent compared to that treated with modified Fe@C NPs.

### 3.3. Histological Analysis of Fe@C Treated Plaques after Implantation

**Figure 5:**
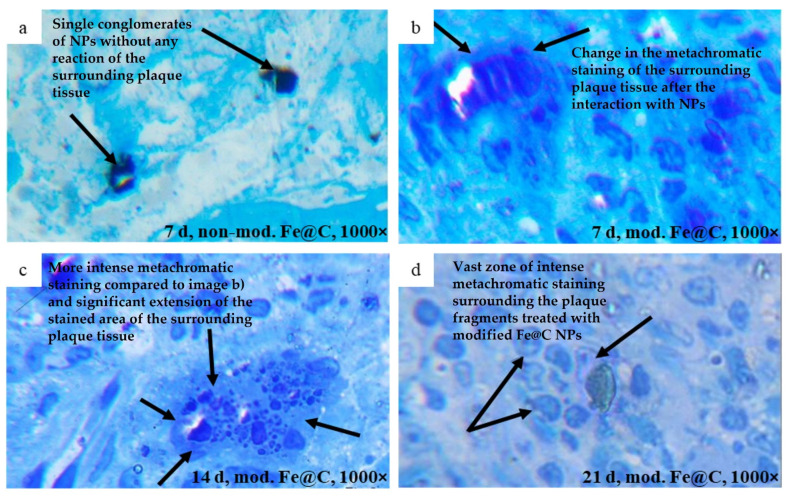
Histomorphological images of human atherosclerotic plaque tissue treated with: (**a**) non-modified Fe@C NPs after seven days subcutaneous implantation in rat; (**b**) modified Fe@C NPs after seven days implantation; (**c**) modified Fe@C NPs after 14 days implantation; (**d**) modified Fe@C NPs after 21 days implantation. The histological structures were obtained using light microscopy and studied in semi-thin sections, dyed with 1% solution of methylene blue with an optical magnification of 1000×. The arrows indicate sample areas, which were located in direct contact with Fe@C NPs and their agglomerates, respectively. Formation of dark blue tissue reaction zones in (**b**–**d**) indicated by arrows are present for modified Fe@C but not after contact of plaques with non-modified Fe@C NPs. Micrographs show expansion of these areas after 14 and 21 days of implantation (highlighted by arrows), indicating specific interaction of chemically modified Fe@C NPs with the structures of atherosclerotic plaques.

Histological analysis was performed to examine the changes in the atherosclerotic plaque tissue caused by the interaction with modified Fe@C NPs and non-modified Fe@C NPs under in vivo conditions. The morphological image of atherosclerotic plaque tissue treated with non-modified Fe@C NPs after seven days of implantation in rat obtained by light microscopy (Figure 5a) showed the presence of Fe@C NPs in the plaque tissue without any visible effect on its structure (highlighted by arrows). The same results related to the non-modified Fe@C NPs were observed after 14 and 21 days of implantation in rats. Conversely, the plaque treatment using modified Fe@C NPs resulted in the first signs of structural plaque changes in the area of its contact with Fe@C NPs, and manifested as a light metachromatic lilac staining of the intercellular substance after seven days of in vivo experiments (Figure 5b, highlighted by arrows). After 14 days of implantation, the metachromatic staining became more intense and the size of the stained area increased significantly (Figure 5c, highlighted by arrows). The 21 days of implantation resulted in the presence of a vast zone of intensive metachromatic staining surrounding the plaque fragments treated with modified Fe@C NPs, as well as a large number of monocytic-macrophage cells of the laboratory animal organism (Figure 5d, highlighted by arrows).

Table 1 presents the results of a quantitative method for assessing the area of metachromatic staining in the structure of atherosclerotic plaques, depending on the time of their contact with chemically modified and non-modified nanoparticles in the subcutaneous tissue of laboratory animals. The results show dynamics of changes in the area of metachromatic staining, which were present in the group with modified Fe@C NPs.

### 3.4. Electron Microscopy Study of Plaques Treated with Modified Fe@C after Implantation

**Figure 6:**
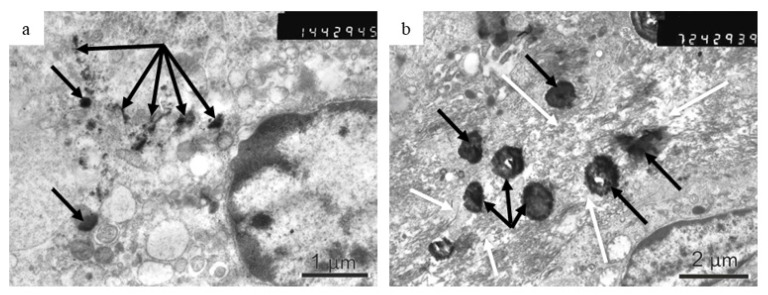
Electron microscopy images of atherosclerotic plaque structure treated with chemically modified Fe@C NPs after 14 days subcutaneous implantation in rats. (**a**) A macrophage of the laboratory rat with modified Fe@C NPs in its cytoplasm (indicated by black arrows). (**b**) Lowered density of collagen (bright areas indicated by white arrows) in the plaque structure in the intercellular space as a result of the interaction with modified Fe@C NPs (dark particles indicated by black arrows). The numbers in the right corner mean the number of EM scan.

To assess the interaction of chemically modified Fe@C NPs and human atherosclerotic plaque tissues on a microscale an EM study was performed. Figure 6 shows an example EM image of a human atherosclerotic plaque treated with chemically modified Fe@C NPs after 14 days of implantation. As can be seen in Figure 6a, a macrophage cell appearing in the cytoplasm and modified Fe@C NPs were subjected to phagocytosis. In Figure 6b, aggregates of Fe@C NPs are located in the intercellular space visible as electron-dense black areas of different sizes. The lowered electron density of the neighboring areas of aggregated modified Fe@C NPs indicates exhaustion of the collagen structure in the place where the Fe@C NPs are situated (shown by white arrows). Figure 6 provides a basis for the assumption that the modified Fe@C NPs can influence the change in the structure of atherosclerotic plaques.

## 4. Discussion

The necessity for efficient mechanisms and materials that could affect the structure of an atherosclerotic plaque remains an urgent issue in the cardiovascular field. Although the growth mechanism and formation of atherosclerotic plaques in human arteries has been studied intensely, the inhibition process of the plaque progression or its destroying is still unsolved [9,22,23]. Attempting to prevent the growth of diagnosed atherosclerotic plaques, before they result in unfavorable outcomes, is one of the main challenges of cardiovascular research [6].

In this study, Fe@C NPs were modified with lipophilic ligands mimicking the surface properties of natural lipoproteins, known to function as cholesterol acceptors. Firstly, the cytocompatibility of the Fe@C NPs in their initial state, as well as after chemical modification, were tested in vitro with HUVEC culture. Secondly, the influence of chemically modified Fe@C NPs on the structure of human atherosclerotic plaques under in vivo conditions during 21 days of implantation in rats was studied using morphometric and histological analysis, as well as EM.

During cell culture, it was seen that chemical modification did not alter the cellular reaction of HUVECs on Fe@C NPs; however, it should be noted that the number of adherent cells after 24 h was significantly reduced for both non-modified Fe@C NPs and chemically modified Fe@C NPs compared to the PS control. Only 10–20% of the 20,000 cells seeded were adherent to the substrate plates coated with NPs. This effect is not unusual, since PS well plates, which are designed for cell adhesion, allow immediate cell attachment and proliferation. This effect should not be considered as too important, as the stainless-steel substrates in the current case were not specifically designed to increase protein adsorption or cell adhesion. Furthermore, the coated steel substrates were placed inside the PS plates, leaving some space around the steel discs. This might allow the cells to slide off onto PS plates during seeding or to migrate during the first 24 h, causing a decrease in adhesion efficiency. Thus, it was more crucial to consider the cell number development from the adherent cells after 24 h as a starting point, giving the cell number development on coated steel substrates. It was suspected that Fe@C NPs lead to better cell adhesion and more cell-typical development than plain PLA coatings. This was especially observed for both higher concentrated modified Fe@C NPs using fluorescence microscopy. This was observed earlier by Ying and Hwang for carbon-coated iron nanoparticles, where the size of the nanoparticles (10 and 50 nm) did not impact toxicity, but the surface coating altered the effects of cytotoxicity significantly [24]. Their experimental studies with carbon-coated iron NPs suggested that the NPs are tolerated by cells even for large concentrations when they are well dispersed or in small clusters. Thus, it is complicated to assess the cytotoxicity effects of iron oxide NPs in an immobilized state, which is relevant for the NPs possible application as a stent coating. According to the presented results in Figure 2, the Fe@C NPs modification seems to be more influencing than the percentage of Fe@C NPs used in terms of cytocompatibility. Fe@C containing samples did not cause significant differences between them in terms of compatibility and seem to follow the same trend during the seven days.

It would be important to model the exposure of the implanted atherosclerotic plaques to the blood flow to mimic better the real in vivo conditions for the proposed application. However, blood flow could lead to the quicker removal of nanoparticles from the plaque surface and inhibition of the observed plaque destroying effect. The proposed subcutaneous model of in vivo test provided static conditions, under which the described impact of nanoparticles on plaques could occur more quickly, providing a specific accelerating effect.

A significant measure of the therapeutic outcome of stents is the pace of re-endothelialization [25]. The rate of endothelization following the injury created by the stent deployment is a critical determinant since this induces a cascade of pro-inflammatory events that result in smooth muscle cell hyperproliferation and usually areas of rapid re-endothelialization present restenosis [26]. Busch et al. investigated the impact of various biodegradable polymers, including PLA, on the growth of vascular endothelial cells, platelets, and smooth muscle cells [27]. Unfortunately, they could not identify a material that enhances endothelial proliferation while at the same time reducing the proliferation of smooth muscle cells. For this reason, in the present study, the efficiency of the Fe@C NPs on plaques during in vivo testing was the first subject of direct testing (Fe@C powdered plaques) without using the PLA coating component.

The penetration ability of the chemically modified Fe@NPs into the inner structure of the plaque tissue observed using morphometric analysis correlates with results demonstrated in [4], where very small superparamagnetic iron oxide NPs were used for plaque visualization using magnetic resonance imaging. Chemically modified Fe@C NPs are lipophilic comparing to the non-modified Fe@C NPs employed in this study. Lipophilicity is known to be responsible for a good adsorption, allowing to use lipophilic structures as drug-delivery molecules [28]. Our results demonstrate the importance of lipophilicity as a property enabling NPs to penetrate the inner structure of human atherosclerotic plaques. Additionally, the penetration ability of the NPs into plaques is influenced by their size, surface chemistry, and shape [29]. The observed results demonstrate a sufficient ability of the chemically modified Fe@C NPs to penetrate the plaque structures offering the potential to be applied as an anti-plaque agent. To confirm these properties, further research focusing on the microscale investigations and additional in vivo tests using other animal models as well as longer implantation times has to be performed.

Consisting of fibrous tissues, lipids, and calcification areas, atherosclerotic plaques represent a very complex composite which content can vary in different parts of the vasculature [30]. The accumulation of the chemically modified Fe@C NPs and the inspected lowering density of the collagen in the plaque tissue, situated in intercellular space, as a result of in vivo incubation with chemically modified Fe@C NPs was obtained by histological analysis. Additional histological and immunohistochemical tests could be necessary to better understand the mechanism of the structural changes. Special interest was aroused by the changes in the morphological tissue of an atherosclerotic plaque at the site of immediate contact with modified Fe@C NPs. It has been shown that, in comparison to lipids, Fe@C NPs enter a biochemical reaction with the structure of human atherosclerotic plaques, which is manifested through a change of staining color and results in lower density collagen fibers. This result correlates within [31] reported interaction of iron oxide NPs with collagen structure demonstrating the ability of collagen to absorb and associate with NPs. This outcome lets us assume that Fe@C NPs can influence the morphological composition of human atherosclerotic plaques. The discovered effect cannot be considered as an immune reaction of the host organism to the transplant because in all experiments the same volume of material has been implanted and the observed reaction was induced only by plaques treated with chemically modified Fe@C NPs.

The EM images, obtained in this work, confirm the presence of chemically modified Fe@C NPs in the macrophage cytoplasm of a laboratory rat, which would probably result in cholesterol efflux from lipid-laden macrophages and its further metabolization. A careful study of the plaque samples revealed that the Fe@C NPs are recognized by the host macrophages, which may surround the separately situated large nanoparticles and their smaller fragments will undergo phagocytosis. Macrophages were reported to metamorphose free cholesterol to cholesteryl ester if they take up and degrade more lipoprotein-derived cholesterol than they can excrete [22]. HDL lipoprotein can remove cholesterol from macrophages and retard or reverse foam cell formation, serving as an acceptor for free cholesterol [22]. Thus far, we can assume that the results demonstrated in this study indicate the possible application of chemically modified biomimetic Fe@C NPs for targeting the macrophages, which are the most prevalent cell type in atherosclerotic plaques. To confirm and better understand this fact, further special experiments with macrophages are necessary. Due to the active involvement in intra-plaque cholesterol homeostasis, inflammation, and extracellular matrix degradation, macrophages have a strong influence on plaque development and progression [14].

HDL, the smallest of the lipoprotein particles having a size in the range of 5 to 17 nm [14], is a natural nanoparticle that exhibits an intrinsic affinity for atherosclerotic plaque macrophages. The exploitation of this property by using artificial nano agents, for example, poly(lactic-co-glycolic acid) (PLGA) nanoparticles studied by Sanchez-Gaytann et al. [9] were shown to be an effective approach to affect the atherosclerotic plaques. They showed that their PLGA-HDL technology is predominantly associated with macrophages within the aorta, with this uptake being key for future anti-inflammatory therapies in atherosclerosis, since inflammatory macrophages contribute to atherosclerosis aggravation and may degrade the extracellular matrix by secreting destructive proteases [22]. These results are consistent with previous findings shown by us and other researchers presenting that NPs target atherosclerotic plaques and accumulate in plaque macrophages [19,22]. Chen et al. provide a detailed review of the HDL mimicking NPs for atherosclerosis treatment highlighting the trials to prevent atherosclerosis progression using HDL mimicking NPs only at the early stage of atherosclerosis [32]. Only a few publications investigated the effect of HDL mimicking NPs on the real atherosclerotic plaques [10] while the most of articles are focused on the development of HDL mimicking NPs as contrast agents for early detection of vulnerable plaques. Many research groups over the past few years have shown that long-circulating NPs loaded with anti-inflammatory drugs accumulate within atherosclerotic lesions and induce local anti-inflammatory effects [5] and that NP translocation in the plaque occurs at sites of increased permeability, in the proximity of disrupted endothelium, providing good accessibility for nanomedicines to an atherosclerotic plaque [14]. The results of our study demonstrate for the first time the application of chemically modified Fe@C NPs as antiatherosclerotic agents able to cause structural changes of the human plaque tissue. The experiments are based on the multiple sample analysis providing a reliable statistical result. The results are patented by the authors [33]. To confirm the anti-atherosclerotic effect of the proposed nanomaterial and explore its practical application in stents, further research is needed.

## 5. Conclusions

The application of biomimetic nanomaterials for atherosclerosis treatment seems to be a promising therapeutic approach discussed in the scientific community. Prevailing research aims at the oral administration of NPs, related enhancement of their oral adsorption, quick and effective delivery to plaques, and bioavailability, as well as on the improvement of imaging methods enabling plaque visualization by magnetic resonance imaging. Among different types of HDL-mimicking NPs described in the literature [13], Fe@C NPs have shown promising potential to be used for destroying atherosclerotic plaques being investigated in contact with native human atherosclerotic plaques under in vivo conditions. Being implanted into rats for 21 days, the applied Fe@C NPs did not cause any cytotoxic effect and have shown good in vitro biocompatibility. Taking into consideration the necessity to provide a long-term interaction between plaque and nanomaterial, we suggest the application of Fe@C NPs as an active substance of a biodegradable stent coating, where degradation would provide a controlled and long-term release of the NPs ensuring their interaction with plaques. The performed study can be considered as basic research for further investigations.

## 6. Patents

Akhmedov SD, Afanasiev SA, Filimonov VD, Postnikov PS, Trusova ME, Karpov RS. Agent for the selective adjustment of blood lipids. US9,789,134 B2., 17.10.2017

## Figures and Tables

**Table 1:** Quantitative estimation of metachromatic staining in the structure of atherosclerotic plaques, depending on the time of their contact with chemically modified and non-modified NPs in the subcutaneous tissue of laboratory animals.

Duration of implantation in laboratory animals (10 animals for every group), days	7	14	21
Non-modified Fe@C NPs	no staining	no staining	no staining
Modified Fe@C NPs with the area of metachromatic staining, µm^2^	1988 ± 246	2700 ± 174	1082 ± 201

## Data Availability

Not applicable.
